# 吉非替尼致急性肺损伤——附1例报道

**DOI:** 10.3779/j.issn.1009-3419.2011.02.12

**Published:** 2011-02-20

**Authors:** 莲芳 倪, 新民 刘, 莉 高

**Affiliations:** 1 100034 北京，北京大学第一医院老年科 Department of Geriatrics, Peking University First Hospital, Beijing 100034, China; 2 100034 北京，北京大学第一医院放射科 Department of Radiology, Peking University First Hospital, Beijing 100034, China

吉非替尼是一种口服的表皮生长因子受体酪氨酸激酶抑制剂（epidermal growth factor receptor-tyrosine kinase inhibitor, EGFR-TKI），能与三磷酸腺苷（adenosine triphosphate, ATP）竞争酪氨酸激酶结构域中的ATP结合位点，抑制其磷酸化，从而阻断肿瘤细胞信号传导通路，抑制肿瘤细胞的生长、转移。作为第一个用于晚期非小细胞肺癌（non-small cell lung cancer, NSCLC）治疗的分子靶向药物，吉非替尼在亚裔、女性、非吸烟、腺癌等优势人群中的作用已经多个国际多中心临床试验证实。因其应用方便，副作用相对较小，对优势人群尤其是*EGFR*基因突变人群的有效率高，得到广大医生和NSCLC患者的认可。然而，随着临床的广泛应用，吉非替尼少见但极其严重的副作用急性肺损伤（acute lung injury, ALI）逐渐引起关注。现报道1例应用吉非替尼出现ALI的病例，以提高临床医生的认识。

## 1 临床资料

患者，男性，76岁，主诉发现肺部阴影2个月于2009年8月入院。2个月前患者因活动后气短于外院查CT肺血管成像（CT pulmonary angiography, CTPA）时发现左上肺团块影，1个月前查PET-CT提示左肺上叶尖段高代谢活性结节，考虑恶性病变。1个月前开始出现低热、四肢水肿症状，4年来体重下降15 kg。病程中无盗汗、胸痛、咯血症状，无宠物、鸟类接触史。既往：慢性阻塞性肺病史20余年，吸烟40余年，20支/天。入院体检：T 38 ℃，P 92次/分，R 18次/分，BP 120/60 mmHg。神志清，浅表淋巴结未及肿大，颈静脉怒张，桶状胸，双侧语颤减弱，左下肺叩诊浊音，左下肺呼吸音低，双肺未闻及明显干、湿啰音，心界不大，心律齐，各瓣膜听诊区未闻及杂音。腹部查体无异常，四肢可凹性水肿，双侧对称。可见杵状指。

入院后给予抗感染治疗，查血常规：WBC 6.01×10^9^/L，NE 76.2%，HGB 104 g/L，PLT 272×10^9^/L。尿便常规正常。肝肾功能无明显异常，ESR 66 mm/h，动脉血气（FiO_2_ 21%）PH7.484，PCO_2_ 34 mmHg，PO_2_ 68.2 mmHg。胸部增强CT（图 1A，图 1B）：左肺上叶尖后段不规则软组织密度占位，大小约4.8 cm ×4.5 cm×4.9 cm，病灶浅分叶，边缘不规则，与胸膜宽基底相连，平扫Ct值36 Hu，增强及延迟后Ct值71 Hu、70 Hu，强化不均匀，伴远段阻塞性肺炎，考虑周围型肺癌可能大；双肺慢支肺气肿改变，左下胸膜增厚，左侧少量胸腔积液。行CT引导下经皮肺穿刺，肺活检病理报告：左肺上叶穿刺少许肺组织，其内可见中分化腺癌浸润。全身浅表淋巴结B超示双侧腹股沟多发肿大淋巴结，呈靶环状。骨扫描：四肢长骨皮质血运丰富，代谢旺盛灶，结合临床符合肺性骨病表现，考虑肿瘤分期Ib期，T2aN0M0。肺功能：通气功能严重减退属阻塞型障碍，FEV1 46%，FEV1/FVC 42.46%，弥散功能严重减退，TLCO占预计值26.9%，TLCO/VA占预计值32.8%。心脏彩超：左心室整体收缩功能正常，LVEF＞50%（simpson法）。

**1:**
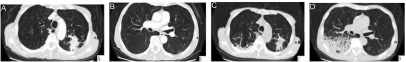
应用吉非替尼前后肺CT变化。A和B：吉非替尼治疗前，左肺上叶尖后段不规则软组织占位；C和D：吉非替尼治疗后，右肺上叶间质病变伴渗出。 Chest CT scan results comparison before and after gefitinib therapy. A and B: before gefitinib therapy; C and D: after gefitinib therapy.

经积极抗感染治疗后，患者体温正常，喘憋有所好转，但一般情况差，肺功能严重减退，围手术期风险大，家属不同意手术治疗。患者体能状态评分（performance status, PS）3分，化疗耐受性差，获益可能性小，与患者家属商议后同意行靶向药物治疗（因活检肺组织少，未行EGFR基因突变检测）。患者于9月11日（图 2A）始予吉非替尼（易瑞沙）250 mg/d口服。9月15日出现高热、喘憋，体温38 ℃-39.4 ℃，血气（FiO_2_ 21%）PH7.48，PCO_2_ 30.8 mmHg，PO_2_ 57.9 mmHg。9月17日血常规：WBC 11.27×10^9^/L，NE 82.6%，HB 104 g/L，PLT 258 ×10^9^/L，胸片（图 2B）示右上肺大片渗出，考虑不排除感染。予哌拉西林舒巴坦（一君）（9月17日-9月22日）抗感染无效，改用美罗培南（美平）（9月22日开始）联合替考拉宁（他格施）（9月24日开始）抗感染治疗，病情无明显好转。9月23日肺CT（图 1C，图 1D）示左上叶软组织肿块较前增大，右肺上叶间质病变伴渗出。痰培养阴性，降钙素原阴性，真菌G、G-M试验阴性，考虑不除外易瑞沙致急性肺损伤。于9月23日停用易瑞沙，继续抗感染治疗。因患者一般情况差，患者及家属不同意行气管镜检查以明确病因。9月27日胸片（图 2C）示右肺上叶后段大片状影，提示病变仍进展，遂予甲基强的松龙80 mg/d静滴。次日体温正常，右肺啰音减少，动脉血气（FiO_2_ 21%）PH7.471，PCO_2_ 37.7 mmHg，PO_2_ 63.5 mmHg。9月30日胸片（图 2D）示右上叶后段大片状影明显吸收并密度减低，1周后（10月4日）改为强的松龙40 mg/d口服，每周减量5 mg，胸片提示右上叶后段大片状影基本吸收。

**2:**
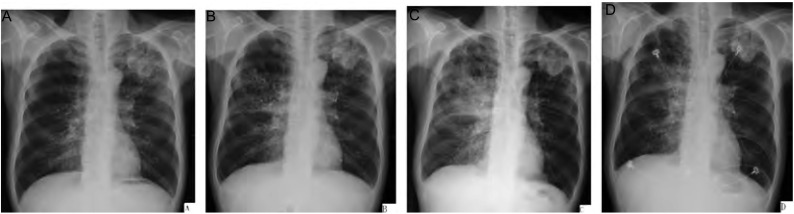
用吉非替尼前后及用激素前后胸片变化。A：吉非替尼治疗前，左上肺团块影；B和C：吉非替尼治疗后，右上肺渗出；D：糖皮质激素治疗后，右上肺渗出明显吸收。 Chest X-ray results comparison before and after gefitinib or glucocorticoids therapy. A: before gefitinib therapy; B and C: after gefitinib therapy; D: after glucocorticoids therapy.

## 2 讨论

NSCLC约占肺癌总数的80%，由于缺乏有效的早期诊断方法，70%-80%的肺癌患者就诊时已为晚期，失去根治性手术切除机会。而针对晚期NSCLC，化疗新药的疗效似已达到平台期。分子靶向药物的出现为肺癌的治疗带来了新的希望和手段，以吉非替尼（易瑞沙）和厄罗替尼（特罗凯）为代表的TKI是目前被多国批准并被广泛应用于进展或难治性NSCLC的小分子靶向药物。事实上，对于不能耐受手术、放化疗的老年肺癌患者，靶向治疗常是适用的抗肿瘤方案。

吉非替尼于2002年首先在日本上市，2005年在中国上市，获批用于治疗既往接受过化疗的局部晚期或转移性NSCLC。最常见的不良反应为腹泻、皮疹、瘙痒、皮肤干燥和痤疮，一般发生于服药后1个月内，通常是可逆的^[1]^。随着临床的广泛应用，吉非替尼导致的肺损伤逐渐引起关注。日本学者Okamoto等^[2]^报道了首例因服用吉非替尼而导致严重间质性肺炎死亡的病例。患者给药8天后即出现呼吸困难，尽管给予大剂量类固醇激素治疗，仍然在给药13天后死亡。在日本吉非替尼引发间质性肺病（interstitial lung disease, ILD）的发生率较高，文献^[3]^报道发生率为3.2%-5.8%。在美国，接受吉非替尼治疗的患者ILD的发生率约为1%^[4]^。国内对于吉非替尼引发的ILD仅有个案报道，没有发病率的统计^[5]^。吉非替尼引发的ILD一旦发生，危险性大、死亡率高。文献^[3]^报道日本吉非替尼引发ILD的死亡率在30%-60%。

吉非替尼导致ALI的机制尚不明确。有专家^[6]^推测是因为对EGFR的抑制作用妨碍了肺部损伤的正常修复，从而加重了肺部损伤，并使免疫炎症反应失控，导致ALI的发生。也有报道^[7]^认为氧化应激参与了间质病的发生，研究发现吉非替尼引起的ILD患者血清中硫氧还蛋白水平增高，但具体机制还有待进一步研究。多项研究进行的多因素分析提示男性、既往有肺纤维化、PS评分差、有放疗史是ALI发生的独立影响因素^[3]^，这可以为临床制定治疗决策提供一些参考。

吉非替尼引起的ALI的临床表现与其它急性肺损伤类似。一般在用药后4周内出现，表现为活动后气短、干咳，伴或不伴发热，停药后病情仍可进展。影像学表现为弥漫性间质病变或磨玻璃样改变。病理提示弥漫性肺泡损伤，肺泡上皮肿胀，肺泡间隔增厚，伴淋巴细胞、中性粒细胞、嗜酸性粒细胞浸润，伴或不伴透明膜形成。病原学检查阴性。血清学上可有LDH、KL-6、SP-D、SP-A升高。吉非替尼引起的ALI的治疗首先是停药，一般需要应用糖皮质激素，文献报道^[8]^一般用甲基强的松龙静脉点滴1 g/d，连续应用3天，继以强的松龙60 mg/d口服，每周减量10 mg，直至减停。一般数日内症状缓解，血气、影像学可以得到改善，表现为嗜酸细胞性肺炎者停药后即可好转^[9]^。本文病例在出现肺损伤给予停药后病情仍进展，给予甲基强的松龙静脉点滴80 mg/d，连续应用1周后影像学明显好转。可见，在激素的用量上可根据临床情况确定，以减少大量激素所带来的副作用。

对于很多晚期NSCLC患者，尤其是放化疗不能耐受或失效者，靶向治疗可能是唯一的抗肿瘤治疗方案，即使出现了严重的副作用，在对症处理好转后，仍存在进一步抗肿瘤治疗的问题。关于肺损伤后的再次用药，文献报道不多。Takamochi等^[10]^报道了1例56岁男性，肺腺癌IIIb期，吸烟，术后辅助化疗（卡铂+紫杉醇）2周期，17个月后出现全身（肺、骨、脑）转移，改用吉非替尼250 mg/d+全脑照射，14天后影像学提示肿瘤明显好转。但用药至45天，患者出现乏力、气短，影像学提示间质性肺浸润，考虑吉非替尼导致的ILD，予停药，并用甲基强的松龙1 g/d静滴，连续3天，后改为50 mg/d，每周减10 mg，1月后ILD好转。停药5月后肿瘤进展，再次用吉非替尼125 mg/d治疗仍有效，未再出现ILD。而Suzuki等^[11]^则报道1例再次用药后出现更严重ILD的病例。患者为59岁男性，肺腺癌Ⅳ期，吸烟，PS为1分，卡铂+紫杉醇一线化疗失败，改用吉非替尼250 mg/d二线治疗，用药后恶性胸水及血CEA均有改善，但23天后出现高热、气短，影像学提示ILD，停药并使用甲基强的松龙后ILD好转。但1月后肿瘤进展，再次用吉非替尼250 mg/d治疗，连用1周，歇2周，仍有效，第3个循环时再次出现ILD，且较前更重，但激素治疗仍有效。如何预测是否会再出现ILD，目前尚不清楚，需要有更多的临床观察来得出更进一步的结论。因此，笔者认为，当病情需要再次用药的时候，须充分与患者及家属交代再次发生ILD的可能性，否则应避免再次应用。

综上所述，吉非替尼引起的ALI一旦发生，往往较凶险，病死率高，应引起广大临床医生的重视。对于门诊处方吉非替尼，则应充分向患者交代风险，一旦出现相关呼吸系统症状，立即就医，及时行影像学检查，以期早期发现、诊断和及时处理，以减少死亡率。

## References

[1] Jiang H (2009). Overview of gefitinib in non-small cell lung cancer: an Asian perspective. Jpn J Clin Oncol.

[2] Okamoto I, Fujii K, Matsumoto M (2003). Diffuse alveolar damage after ZD1839 therapy in a patient with non-small cell lung cancer. Lung Cancer.

[3] Nakagawa M, Nishimura T, Teramukai S (2009). Interstitial lung disease in gefitinib-treated Japanese patients with non-small cell lung cancer-a retrospective analysis: JMTO LC03-02. BMC Res Notes.

[4] Shan NT, Kris MG, Pao W (2005). Practical management of patients with non small-cell lung cancer treated with gefitinib. J Clin Oncol.

[5] Zhen D, Shan NZH, Ping DZD (2004). Monotherapy with gefitinib (Iressa) on patients with advanced non-small cell lung cancer and resistant to prior chemotherapy. Chin J New Drugs.

[6] Wu HB (2006). The clinical manifestation of advere reactions to gefitinib and their management. Adverse Drug React J.

[7] Sakuma K, Nakamura H, Nakamura T (2007). Elevation of serum thioredoxin in patients with gefitinib-induced interstitial lung disease. Intern Med.

[8] Seto T, Seki N, Uematsu K (2006). Gefitinib-induced lung injury successfully treated with high-dose corticosteroids. Respirology.

[9] Kitajima H, Takahashi H, Harada K (2006). Gefitinib-induced interstitial lung disease showing improvement after cessation: disassociation of serum markers. Respirology.

[10] Takamochi K, Suzuki K, Bashar AH (2007). Readministration of gefitinib in a responder after treatment discontinuation due to gefinitib-related interstitial lung disease: a case report. J Med Case Reports.

[11] Suzuki M, Asahina H, Konishi J (2008). Recurrent gefitinib-induced interstitial lung disease. Inter Med.

